# Sexually transmitted infections and HIV RNA levels in blood and anogenital compartments among Thai men who have sex with men before and after antiretroviral therapy: implication for Treatment as Prevention programme

**DOI:** 10.1002/jia2.25186

**Published:** 2018-09-18

**Authors:** Nittaya Phanuphak, Supanit Pattanachaiwit, Tippawan Pankam, Nipat Teeratakulpisarn, Parinya Chamnan, Panita Pathipvanich, Suchart Thongpaen, Siriporn Nonenoy, Jureeporn Jantarapakde, Supabhorn Pengnonyang, Deondara Trachunthong, Thanthip Sungsing, Kittiyaporn Parasate, Sriprai Seeneewong Na Ayutthaya, Ketmookda Trairat, Kanitta Pussadee, Cheewanan Lertpiriyasuwat, Praphan Phanuphak, the Thai MSM/TG Test and Treat Study Group

**Affiliations:** ^1^ PREVENTION Thai Red Cross AIDS Research Centre Bangkok Thailand; ^2^ Tawanghin Community Medical Care Center‐Sanpasitthiprasong Hospital Ubonratchathani Thailand; ^3^ Lampang Hospital Lampang Thailand; ^4^ Mahasarakam Hospital Mahasarakam Thailand; ^5^ HIV‐NAT Thai Red Cross AIDS Research Centre Bangkok Thailand; ^6^ Department of Disease Control Ministry of Public Health Nonthaburi Thailand

**Keywords:** anogenital, HIV RNA, men who have sex with men, sexually transmitted infections, treatment as prevention

## Abstract

**Introduction:**

Sexually transmitted infections (STIs) are common among HIV‐positive men who have sex with men (MSM). There have been concerns that undiagnosed and untreated STIs could undermine efforts to use antiretroviral therapy (ART) for prevention due to genital secretion infectiousness. We evaluated the correlation between STIs and HIV RNA in anogenital compartments among HIV‐positive MSM before and after ART.

**Methods:**

MSM participants newly diagnosed with HIV were offered ART regardless of CD4 count during November 2012 to November 2015. Syphilis serology, oropharyngeal swab, rectal swab, urine collection for gonorrhoea and chlamydia nucleic acid amplification testing, and HIV RNA measurement in blood, semen and rectal samples were performed at baseline, 12 and 24 months thereafter.

**Results:**

Of 143 HIV‐positive MSM, 16.1% had syphilis, 23.1% had gonorrhoea and 32.8% had chlamydia at baseline. Participants with STIs at baseline had higher median HIV RNA levels in blood plasma (*p* = 0.053), seminal plasma (*p* = 0.01) and rectal secretions (*p* = 0.002) than those without STIs. Multivariate models identified HIV RNA 100,000 to 500,000 (OR 6.74, 95% CI 2.24 to 20.28, *p* = 0.001) and >500,000 (OR 9.39, 95% CI 1.08 to 81.72, *p* = 0.04) copies/mL in blood, CD4 count <350 cells/mm^3^ (OR 4.20, 95% CI 1.05 to 16.70, *p* = 0.04) and having any STIs (OR 2.62, 95% CI 1.01 to 6.80 *p* = 0.047) to be associated with detectable (>40 copies/mL) seminal plasma HIV RNA. Having chlamydia at any sites (OR 3.17, 95% CI 1.07 to 9.44, *p* = 0.04) was associated with detectable rectal HIV RNA. Incidences of syphilis, gonorrhoea and chlamydia were 13.4, 16.4 and 18.1 per 100 person‐years respectively. Nine participants had detectable HIV RNA (five in blood, one in semen, two in rectal samples and one in both blood and rectal samples) at 12 and/or 24 months after ART.

**Conclusions:**

STIs were extremely common among HIV‐positive MSM prior to and after ART. ART effectively reduced HIV RNA in all compartments. The correlation between STIs and anogenital HIV RNA, especially prior to ART and likely until complete HIV RNA suppression from ART is achieved, points to the importance of integrating asymptomatic STIs screening into Treatment as Prevention programme for MSM.

## 1 Introduction

Sexually transmitted infections (STIs) have been associated with biological risk for HIV transmission and acquisition 1. For men who have sex with men (MSM), these STIs can occur in the oropharyngeal, urethral and rectal compartments. Undiagnosed and untreated STIs among MSM are common in many settings where access to STIs diagnosis and treatment is limited by the hidden and stigmatized nature of male same‐sex behaviour and providers’ lack of knowledge and skills in STIs 2, 3. In addition, costs of screening and treatments are major obstacles for MSM access to STIs services. Among MSM in the US, higher rates of undiagnosed and untreated syphilis have been associated with higher rates of HIV infection 4. Rectal gonorrhoea was also found to be associated with HIV seroconversion among MSM in many settings 5, 6, 7.

Treatment as Prevention is one of the key strategies to end the AIDS epidemic as a public health threat 8. Countries are focusing their efforts to enhance access to HIV testing and immediate linkage to antiretroviral therapy (ART) among key populations, including MSM, in order to suppress one's HIV RNA so that it becomes untransmittable. However, with high STIs burden among MSM both prior to and after ART, there have been concerns that undiagnosed and untreated STIs among HIV‐positive MSM could undermine efforts to use ART for prevention by increasing genital secretion infectiousness 9.

There has been conflicting evidence regarding the relationship between the presence of anogenital STIs and the detection of HIV RNA in the anogenital compartments, with and without ART. A case series in men suggested that treatment of asymptomatic urethral STIs could reduce seminal plasma HIV RNA 10. One small study in MSM, however, reported that rectal HIV RNA was correlated with plasma HIV RNA and ART, but not with the presence of rectal STIs 11. A recent meta‐analysis also found that the average effect of STIs co‐infection on HIV RNA in individuals on ART is less than 1 log_10_ difference, and is thus unlikely to decrease the effectiveness of treatment as prevention 12.

Previous studies are limited in the small number of anogenital samples available for concurrent STIs testing and HIV RNA measurement and hold different time durations after ART initiation. None of these studies were conducted among Asian HIV‐positive MSM. We, therefore, studied the correlations between STIs and anogenital HIV RNA among HIV‐positive MSM before and over a 24‐month period after ART in the “Test and Treat” Demonstration Project in Thailand. This information is very important as a key parameter to be considered when modelling the impact of HIV prevention strategies on HIV transmission among MSM population.

## 2 Methods

### 2.1 Enrolment and follow‐up of study participants

We utilized data from Thai HIV‐positive MSM participating in the Test and Treat Demonstration Project between November 2012 and November 2015. This was an observational cohort study to examine the feasibility and acceptability of HIV testing and ART initiation regardless of CD4 count among Thai MSM and transgender women (TG). During the first 24 months of the study, ART initiation at any CD4 count was not part of the routine clinical practice as the Thailand National HIV Guidelines had only launched this recommendation in late October 2014 13. The project also aimed to examine HIV RNA suppression among HIV‐positive participants, changes in risk behaviour and rates of STIs over the study period (clinicaltrials.gov identification NCT01869595).

Thai men and TG, aged 18 years and above, who were not known to be HIV‐positive, engaged in unprotected rectal intercourse with men at least one time or had at least three male sex partners in the last six months, were enrolled from the Thai Red Cross Anonymous Clinic in Bangkok, Sanpasitthiprasong Hospital in Ubonratchathani, Lampang Hospital in Lampang and Mahasarakam Hospital in Mahasarakam, Thailand. After being given informed consent, all participants received HIV testing. An online self‐administered questionnaire was used to assess sexual risk behaviours, including condom use, number of sex partners and drug use behaviours, with these factors considered at enrolment and later every six months. HIV‐positive participants were offered ART initiation regardless of CD4 count. Those who accepted immediate ART were scheduled to have follow‐up visits at weeks two, four, eight, twelve and every three months after that until month 24. Those who did not accept immediate ART were scheduled every six months until month 24. All HIV‐positive participants received risk reduction counselling along with condoms and lubricants at all study visits.

The study was approved by the Office of Joint Research Ethics Committees, the ethics committee of the Institute for the Development of Human Research Protections at the Ministry of Public Health, and the institutional review boards of Chulalongkorn University, Sanpasitthiprasong Hospital, Lampang Hospital and Mahasarakam Hospital. All participants gave informed consent.

### 2.2 CD4 count and HIV RNA measurement

CD4 count was measured at baseline and every six months. HIV RNA measurement (Abbott RealTime HIV‐1, Abbott Molecular Inc., IL, USA) in blood was performed at baseline and every six months, and in seminal plasma and rectal secretions at baseline, month 12 and month 24. Semen and rectal secretions were collected in the clinic. Seminal plasma was prepared from semen collected at baseline, month 12 and month 24. Semen sample was centrifuged at 2330 *g* for 10 minutes at room temperature. Then, 1 mL of supernatant was aliquoted, incubated at 56°C for 30 minutes and used for HIV RNA measurement. Rectal secretions were collected using a sponge with drawstring (Merocel^®^ Schindler Ear Packing, Meditronic Xomed, Inc., Florida, USA). A quantity of 1 mL of extraction buffer was added to the sponge, followed by ten‐ to fifteen‐minute incubation and five‐minute centrifugation at 2385 *g* at 4°C. The collection and quantification of rectal HIV RNA were validated using external quality assurance samples from the Department of Medical Sciences, Thai Ministry of Public Health, and demonstrated a variation of ± 0.5 log_10_ copies/mL in measurement. Blood contamination in rectal samples was excluded using the Hema‐Screen^®^ Lab Pack (Stanbio Laboratory, EKF Diagnostics, TX, USA). Genotypic resistance testing was performed on blood samples from all participants at baseline and from participants with blood plasma HIV RNA above 1000 copies/mL after at least six months of ART.

### 2.3 STIs screening

Syphilis serology, using rapid plasma reagin (RPR) or venereal disease research laboratory (VDRL) screening, followed by treponema pallidum haemagglutination assay (TPHA) confirmation, and gonorrhoea and chlamydia nucleic acid amplification testing (NAAT, Abbott Real Time CT/NG, Abbott Molecular Inc.) of oropharyngeal swab, rectal swab and urine were performed at baseline, month 12 and month 24. Participants diagnosed with any of these STIs received treatment accordingly at the study sites.

### 2.4 ART and adherence assessment

Risks and benefits of ART regardless of CD4 count were discussed with HIV‐positive participants. The first‐line ART regimen included tenofovir disoproxil fumarate (TDF) 300 mg, lamivudine (3TC) 300 mg or emtricitabine (FTC) 200 mg and efavirenz (EFV) 600 mg taken once daily. Other standard regimens were available for participants who could not tolerate any component of the first‐line regimens. Adherence to ART was assessed every six months using a self‐completed visual analogue scale (VAS) for HIV‐positive participants on ART.

### 2.5 Statistical analysis

Statistical analysis was conducted with Stata version 14.1 (Statcorp, College Station, TX, USA). The demographic characteristics and behaviour risk information at baseline, month 12 and month 24 were summarized as median (interquartile range, IQR) and number (percentage) for continuous and categorical variables respectively. The baseline prevalence of STIs was calculated together with 95% confidence intervals (CI) according to a binomial distribution. The incidence rate of STIs over 12 months was calculated per person‐time at risk. Person‐time was calculated using time periods between the actual visit dates of participants at risk, and 95% CI around the incidence was estimated assuming a Poisson distribution. HIV RNA levels in different compartments, changes in rates of STIs and risk behaviour from baseline were compared over the 24‐month study duration for HIV‐positive participants using McNemar's test and Wilcoxon signed‐rank test.

At baseline, a binary logistic regression model was used to explore correlation between STIs and anogenital HIV RNA. Assumptions about linearity of continuous covariates such as age, CD4 count, log plasma HIV RNA and number of sexual partners were checked by breaking the variable into quartiles and examining the odds ratio (OR) and 95% CI for each quartile. When these assumptions were not met, categorical groupings were used and adjacent quartiles were collapsed together if appropriate. Baseline covariates associated with outcomes in univariate regression with *p*‐value of <0.10 were included and adjusted for in multivariate regression models.

We assigned the baseline time point for the analyses to the seroconversion visit of participants who acquired HIV during the study period and subsequent time points were gave based on time on ART.

### 2.6 Role of the funding source

The funders of the study had no role in study design, data collection, data analysis, data interpretation or writing of the report. The corresponding author had full access to all the data in the study and had final responsibility for the decision to submit for publication.

## 3 Results

### 3.1 Participant characteristics

Of 492 participants enrolled into the Test and Treat Demonstration Project, HIV infection was diagnosed in 143 participants; 106 at baseline (21.5% prevalence) and 37 over the 24‐month study period (6.4 per 100 person‐years incidence, 95% CI 4.7 to 8.9). All HIV‐positive participants were MSM. The mean (SD) age was 25.8 (6.0) years old (Table 1). Around half had more than one partner in the past month and 73.9% did not use condoms consistently. The median baseline CD4 count was 348 (250 to 462) cells/mm^3^.

**Table 1:** Baseline characteristics of HIV‐positive participants

Characteristics	Overall (N = 143)	HIV‐positive at baseline (n = 106)	HIV seroconversion (n = 37)	*p*‐value
Age (years), mean (SD)	25.8 (6.0)	25.7 (5.3)	26.2 (7.7)	0.66^a^
Age ≥25, n (%)	63 (44.1)	48 (45.3)	15 (40.5)	0.62^c^
Gender, n (%)				
MSM	143 (100)	106 (100)	37 (100)	
Education, n/N (%)				0.63^c^
Less than bachelor degree	59/129 (45.7)	46/98 (46.9)	13/31 (41.9)	
Bachelor degree/above	70/129 (49)	52/98 (49.1)	18/31 (48.7)	
CDC stage, n/N (%)				
A	‐	90/100 (90)	‐	
B	‐	9/100 (9)	‐	
C	‐	1/100 (1)	‐	
HIV RNA				
Blood plasma (n = 137), median (IQR) log_10_ copies/mL	4.9 (4.2 to 5.5)	4.8 (4.2 to 5.5)	5.0 (4.4 to 5.4)	0.53^b^
<40 copies/mL, n (%)	0/137 (0)	0/100 (0)	0/37 (0)	>0.99^d^
40 to 1500 copies/mL, n (%)	2/137 (1.5)	2/100 (2.0)	0/37 (0)	
1501 to 100,000 copies/mL, n (%)	70/137 (51.1)	51/100 (51.0)	19/37 (51.4)	
>100,000 copies/mL, n (%)	65/137 (47.5)	47/100 (47)	18/37 (48.7)	
Seminal plasma (n = 103), median (IQR) log_10_ copies/mL	3.4 (1.6 to 4.2)	3.4 (1.6 to 4.2)	2.0 (1.6 to 4.3)	0.23^b^
<40 copies/mL, n (%)	39/103 (37.9)	33/91 (36.3)	6/12 (50.0)	0.40^c^
40 to 1500 copies/mL, n (%)	11/103 (10.7)	9/91 (9.9)	2/12 (16.7)	
>1500 copies/mL, n (%)	53/103 (51.5)	49/91 (53.9)	4/12 (33.3)	
Rectal secretion (n = 108), median (IQR) log_10_ copies/mL	3.7 (1.6 to 4.4)	3.8 (1.6 to 4.5)	1.6 (1.6 to 3.1)	0.002^b^
<40 copies/mL, n (%)	38/108 (35.2)	30/97 (30.9)	8/11 (72.7)	0.003^d^
40 to 1500 copies/mL, n (%)	3/108 (2.8)	2/97 (2.1)	1/11 (9.1)	
>1500 copies/mL, n (%)	67/108 (62.0)	65/97 (67.0)	2/11 (18.2)	
CD4 cell count (cells/mm^3^), median (IQR)	348 (250 to 462)	328.5 (220 to 436)	416 (308 to 530)	0.007^c^
<350, n (%)	70/137 (51.1)	56/100 (56)	14/37 (37.8)	
350 to 500, n (%)	42/137 (30.7)	32/100 (32)	10/37 (27)	
>500, n (%)	25/137 (18.3)	12/100 (12)	13/37 (35.1)	
Hepatitis B, n/N (%)	13/133 (9.8)	10/97 (10.3)	3/36 (8.3)	>0.99^d^
STIs, n/N (%)				
None	66/137 (48.2)	43/100 (43)	23/37 (62.2)	
Any STIs	71/137 (51.8)	57/100 (57)	14/37 (37.8)	0.046^c^
Syphilis	22/137 (16.1)	21/100 (21)	1/37 (2.7)	0.008^c^
*Neisseria gonorrhoeae*				
Pharyngeal	15/132 (11.4)	12/97 (12.4)	3/35 (8.6)	0.87^d^
Rectal	17/132 (12.9)	16/98 (16.3)	1/34 (2.9)	0.09^d^
Urine	6/132 (4.6)	5/98 (5.1)	1/34 (2.9)	>0.99^d^
Any route	31/134 (23.1)	26/98 (26.5)	5/36 (13.9)	0.12^c^
Chlamydia trachomatis				
Pharyngeal	5/132 (3.8)	4/97 (4.1)	1/35 (2.9)	0.66^d^
Rectal	39/132 (29.6)	32/98 (32.7)	7/34 (20.6)	0.41^d^
Urine	8/132 (6.1)	6/98 (6.1)	2/34 (5.9)	>0.99^d^
Any route	44/134 (32.8)	36/98 (36.7)	8/36 (22.2)	0.11^c^
Number of sexual partners in the past month, median (IQR)	2 (1 to 4)	2 (1 to 4)	2 (2 to 4)	0.25^b^
No sexual partner, n/N (%)	16/134 (11.9)	15/98 (15.3)	1/36 (2.8)	
Single partner, n/N (%)	35/134 (26.1)	28/98 (28.6)	7/36 (19.4)	
Multiple partners, n/N (%)	83/134 (61.9)	55/98 (56.1)	28/36 (77.8)	0.04^c^
Having unprotected sex, n/N (%)	96/130 (73.9)	75/98 (76.5)	21/32 (65.6)	0.22^c^
Drug resistance, n/N (%)				
NRTI	1/129 (0.8)	0/97 (0)	1/32 (3.1)	
NNRTI	5/129 (3.9)	4/97 (4.1)	1/32 (3.1)	
PI	3/129 (2.3)	2/97 (2.1)	1/32 (3.1)	
Any	8/129 (6.2)	6/97 (6.2)	2/32 (6.3)	>0.99^d^
Drug use in the past one month, n/N (%)	49/130 (37.7)	36/98 (36.7)	13/32 (40.6)	0.69^c^
Amphetamine‐type stimulants use, n/N (%)	17/130 (13.1)	12/98 (12.2)	5/32 (15.6)	0.76^d^

IQR, interquartile range; MSM, men who have sex with men; STIs,, sexually transmitted infection; NRTI, nucleos(t)ide reverse transcriptase inhibitor; NNRTI, non‐nucleos(t)ide reverse transcriptase inhibitor; PI, protease inhibitor.

a Two sample t test.

b Mann–Whitney test.

c Chi‐square test.

d Fisher's exact test.

### 3.2 Baseline HIV RNA in blood plasma, seminal plasma and rectal secretions

**Figure 1:**
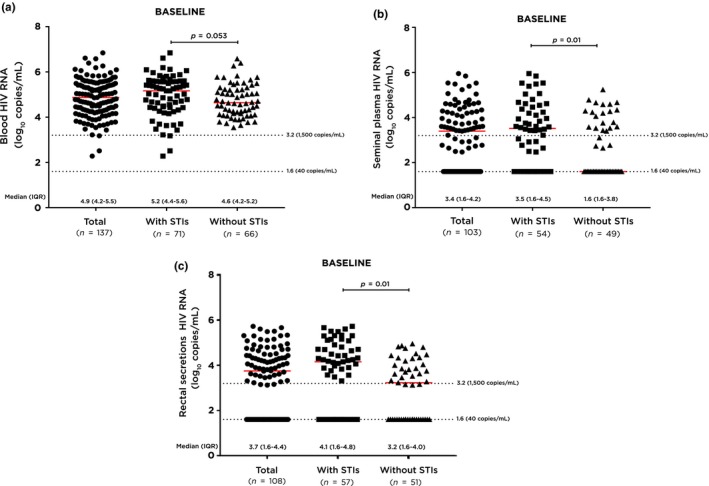
Baseline HIV RNA levels in blood plasma, seminal plasma and rectal secretions among HIV‐positive participants, stratified by status of sexually transmitted infections. (**a**) HIV RNA levels in blood plasma at baseline. (**b**) HIV RNA levels in seminal plasma at baseline. (**c**) HIV RNA levels in rectal secretions at baseline. *p‐values comparing median HIV RNA in blood plasma versus seminal plasma <0.001, blood plasma versus rectal secretions <0.001, seminal plasma versus rectal secretions = 0.09.

Median baseline HIV RNA in blood plasma was 4.9 (4.2 to 5.5) log_10_ copies/mL and 98.5% had blood plasma HIV RNA >1500 copies/mL (Table 1). Median baseline HIV RNA in seminal plasma and rectal secretions were 3.4 (1.6 to 4.2) log_10_ copies/mL and 3.7 (1.6 to 4.4) log_10_ copies/mL respectively. Detectable HIV RNA, defined as a level of 40 copies/mL or higher, was found in 100.0% of blood plasma, 62.1% of seminal plasma and 64.8% of rectal secretions at baseline. Median baseline HIV RNA in blood plasma was significantly higher than those in seminal plasma (*p* < 0.001) and rectal secretions (*p* < 0.001) (Figure 1). Resistant mutations related to nucleoside/nucleotide reverse transcriptase inhibitors (NRTI), non‐nucleoside reverse transcriptase inhibitors (NNRTI) and protease inhibitors (PI) were detected in 0.8%, 3.9% and 2.3% of blood plasma respectively.

### 3.3 Baseline STIs among HIV‐positive participants

Syphilis was found in 16.1% of participants. Gonorrhoeal infection was diagnosed in 23.1% (11.4% in oropharynx, 12.9% in rectum and 4.6% in urethra) while chlamydial infection was diagnosed in 32.8% (3.8% in oropharynx, 29.6% in rectum and 6.1% in urethra).

### 3.4 Associations between STIs and HIV RNA levels in blood and anogenital compartments at baseline

Participants with STIs at baseline had higher median HIV RNA levels in blood plasma (*p* = 0.053), seminal plasma (*p* = 0.01) and rectal secretions (*p* = 0.002) than those without STIs (Figure 1). Participants with HIV seroconversion who provided anogenital samples (n = 12) tended to be less likely to have detectable HIV RNA in these compartments than those who were HIV‐positive at baseline (Table 1). Seroconverters also had a tendency to have less STIs when HIV infection was diagnosed (Table 1). HIV RNA was detectable in seminal plasma in six out of twelve (50.5%) and in rectal secretions in three out of eleven (27.3%, who also had detectable HIV RNA in their seminal plasma) participants. Rectal chlamydia was found among two participants, who both had detectable HIV RNA in their seminal plasma, while no STIs were found among the rest of the seroconverters.

Detectable seminal plasma HIV RNA was associated with HIV RNA 100,000 to 500,000 (OR 6.74, 95% CI 2.24 to 20.28, *p* = 0.001) and >500,000 (OR 9.39, 95% CI 1.08 to 81.72, *p* = 0.04) copies/mL in blood, CD4 count <350 cells/mm^3^ (OR 4.20, 95% CI 1.05 to 16.70, *p* = 0.04) and having any STIs (OR 2.62, 95% CI 1.01 to 6.80 *p* = 0.047) (Table 2). Having chlamydia at any sites (OR 3.17, 95% CI 1.07 to 9.44, *p* = 0.04) was associated with detectable rectal HIV RNA. Rectal gonorrhoea did not show significant correlation with detectable rectal HIV RNA with an OR of 4.54 (95% CI 0.85 to 24.20, *p* = 0.08).

**Table 2:** Univariable and multivariable analysis of factors associated with detectable HIV RNA in anogenital compartments before ART initiation, using all available data in each compartment (n = 103 for seminal plasma and n = 108 for rectal secretion)

Factors	HIV RNA in seminal plasma >40 copies/mL				HIV RNA in rectal secretions >40 copies/mL			
	Univariable		Multivariable		Univariable		Multivariable	
	OR (95% CI)	*p*‐value	OR (95% CI)	*p*‐value	OR (95% CI)	*p*‐value	OR (95% CI)	*p*‐value
Age (years)								
≥ 25	1.05 (0.47 to 2.35)	0.91			0.87 (0.39 to 1.94)	0.74		
CD4 cell count (cells/mm^3^)		0.05		0.12		0.09		0.13
>500	Ref.		Ref.		Ref.		Ref.	
350 to 500	1.90 (0.55 to 6.53)6.533253	0.31	2.98 (0.72 to 12.29)	0.13	1.60 (0.48 to 5.37)	0.45	1.80 (0.47 to 6.91)	0.39
<350	3.90 (1.18 to 12.85)	0.03	4.20 (1.05 to 16.70)	0.04	3.20 (0.99 to 10.34)	0.05	3.57 (0.95 to 13.34)	0.06
HIV RNA in blood plasma (copies/mL)		<0.001		<0.001		0.02		0.23
<100,000	Ref.		Ref.		Ref.		Ref.	
100,000 to 500,000	6.96 (2.49 to 19.43) 19.43325	<0.001	6.74 (2.24 to 20.28)	0.001	2.34 (0.96 to 5.73)	0.06	1.82 (0.68‐ 4.83)	0.03
>500,000	15.30 (1.84 to 127.00)	0.01	9.39 (1.08 to 81.72)	0.04	5.63 (1.16 to 27.31)	0.03	3.39 (0.64 to 18.07)	0.15
Number of sexual partners		0.80				0.64		
No sexual partner	Ref.				Ref.			
Single partner	0.78 (0.20 to 3.02)	0.72			0.56 (0.14 to 2.22)	0.41		
Multiple partners	1.07 (0.31 to 3.70)	0.91			0.57 (0.16 to 2.00)	0.38		
Having unsafe sex	0.76 (0.29 to 2.03)	0.59			0.78 (0.30 to 2.05)	0.62		
Any STIs	2.98 (1.30 to 6.81)	0.01	2.62 (1.01 to 6.80)	0.047	1.94 (0.87 to 4.33)	0.10		
Syphilis	1.90 (0.63 to 5.78)	0.24			1.45 (0.51 to 4.12)	0.47		
Any gonorrhoea	3.00 (1.02 to 8.87)	0.03			1.35 (0.52 to 3.48)	0.53		
Pharyngeal	4.07 (0.85 to 19.47)	0.05			0.62 (0.19 to 2.01)	0.43		
Rectal	2.31 (0.59 to 8.98)	0.20			4.25 (0.91 to 19.98)	0.07	4.54 (0.85 to 24.20)	0.08
Urine	(omitted)^a^				0.83 (0.13 to 5.20)	0.84		
Any chlamydia	2.11 (0.85 to 5.20)	0.10			3.97 (1.47 to 10.73)	0.004	3.17 (1.07 to 9.44)	0.04
Pharyngeal	1.97 (0.20 to 19.60)	0.55			0.55 (0.07 to 4.10)	0.57		
Rectal	1.98 (0.78 to 5.07)	0.14			5.60 (1.78 to 17.58)	<0.001		
Urine	(omitted)^a^				2.94 (0.33 to 26.11)	0.29		

Covariates with *p*‐value <0.10 from the univariable analysis were included in the multivariable analysis. For the seminal plasma model, multicollinearities were identified among any STIs, any gonorrhoea, pharyngeal gonorrhoea and rectal gonorrhoea. Only any STIs was selected into the multivariable model. For the rectal secretions model, multicollinearity between any chlamydia and rectal chlamydia was identified. Only any chlamydia was selected into the multivariable model. CI, confidence interval; STIs, sexually transmitted infection; ART, antiretroviral therapy.

a Omitted as all participants with urethral gonorrhoea or urethral chlamydia had detectable HIV RNA in seminal plasma.

### 3.5 ART initiation and HIV RNA levels in blood and anogenital compartments after 12 and 24 months of ART

Of 143 HIV‐positive participants, 133 were successfully initiated ART after HIV diagnosis. All the participants were started with an ART regimen containing TDF, 3TC and EFV. Two participants had EFV changed to lopinavir/ritonavir due to EFV‐related rashes or central nervous system side effects. There were 25 participants who did not have month 12 visit and 60 who did not have month 24 visit after ART either due to the inability to follow‐up or as a result of having seroconverted and subsequently completing the study prior to those visits. At months 12 and 24 after ART initiation, 70.8% and 78.4% of participants, respectively, showed ≥95% adherence to ART by VAS.

Out of the initial twenty‐two participants who had syphilis at baseline, active syphilis was identified in two out of sixteen who attended month 12 visit and one out of nine at month 24 visit. Urethral gonorrhoea was detected in one out of five at month 12 and zero out of three at month 24, from six participants who had it at baseline. Among eight participants with baseline urethral chlamydia, one out of six had it at month 12 and zero out of three at month 24. Rectal gonorrhoea was found in two out of fifteen at month 12 and one out of seven at month 24, from seventeen participants who had it at baseline. Rectal chlamydia was detected in fourteen out of thirty‐seven at month 12 and three out of twenty at month 24, among thirty‐nine participants who had it at baseline.

HIV RNA became undetectable in the vast majority of participants in all compartments at month 12 and month 24 after ART. HIV RNA in blood plasma was detected in two out of forty‐eight MSM with STIs and three out of sixty‐six MSM without STIs at month 12 (4.2% vs. 4.6%, *p* > 0.99), and two out of twenty‐two MSM with STIs and zero out of forty‐six MSM without STIs at month 24 (9.1% vs. 0%, *p* = 0.19). Detectable HIV RNA in seminal plasma was found in zero out of forty MSM with STIs and one out of fifty‐eight MSM without STIs at month 12 (0% vs. 1.7%, *p* > 0.99), and one out of twenty‐two MSM with STIs and one out of forty‐three MSM without STIs at month 24 (4.6% vs. 2.3%, *p* > 0.99). From rectal secretions, HIV RNA was detected in zero out of thirty‐six MSM with STIs and one out of fifty MSM without STIs at month 12 (0% vs. 2.0%, *p* > 0.99), and zero out of twenty‐three MSM with STIs and zero out of thirty‐eight MSM without STIs at month 24.

**Figure 2:**
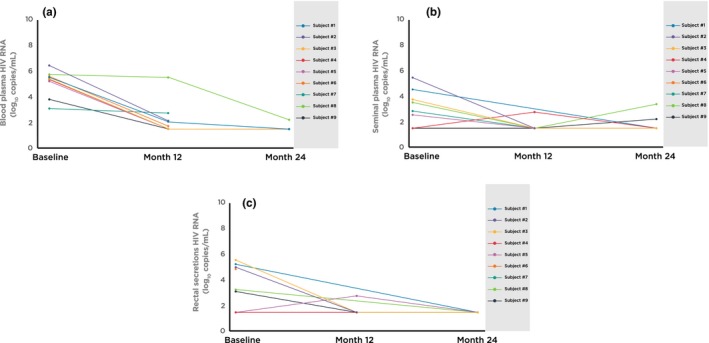
Changes in HIV RNA levels in blood and anogenital compartments among participants with at least one undetectable HIV RNA after antiretroviral therapy. (**a**) HIV RNA levels in blood plasma. (**b**) HIV RNA levels in seminal plasma. (**c**) HIV RNA levels in rectal secretions.

None of the four participants with detectable anogenital HIV RNA after ART (one in semen at month 12, one in rectal secretion at month 12 and two in semen at month 24) had anogenital STIs in the same compartment at those same visits (Table 3 and Figure 2). Among five participants with detectable month 12 HIV RNA in blood, four had treated syphilis (after a diagnosis at baseline) and one had both rectal gonorrhoea and chlamydia at month 12. Of two participants with detectable month 24 HIV RNA in blood, one had rectal gonorrhoea and urethral chlamydia and another one (who also had detectable HIV RNA in semen at this visit) had both rectal gonorrhoea and chlamydia but not any rectal STIs at month 24. This last participant had wild‐type HIV in blood at baseline but at month 12 developed M184V, V106I, Y188I and P225HLP mutations which led to his ART regimen being switched to TDF, FTC and darunavir/ritonavir. He was the only participant with detectable HIV RNA at a level of >1500 copies/mL in blood at month 12 and in semen at month 24.

**Table 3:** Sexually transmitted infections and HIV RNA in blood and anogenital compartments at baseline, months 12 and 24 among participants with at least one undetectable HIV RNA after antiretroviral therapy

	Blood plasma HIV RNA and syphilis						Rectal secretions HIV RNA and rectal STIs									Seminal plasma HIV RNA and urethral STIs								
NO	BL HIV RNA (copies/mL)	BL Syphilis	M12 HIV RNA (copies/mL)	M12 Syphilis	M24 HIV RNA (copies/mL)	M24 Syphilis	BL HIV RNA (copies/mL)	BL Rectal GC	BL Rectal CT	M12 HIV RNA (copies/mL)	M12 Rectal GC	M12 Rectal CT	M24 HIV RNA (copies/mL)	M24 Rectal GC	M24 Rectal CT	BL HIV RNA (copies/mL)	BL Ureth GC	BL Ureth CT	M12 HIV RNA (copies/mL)	M12 Ureth GC	M12 Ureth CT	M24 HIV RNA (copies/mL)	M24 Ureth GC	M24 Ureth CT
1^a^	472,505	R (new)	144	R (treated)	<40	R (treated)	209,681	Neg	Neg	ND	Pos	Pos	<40	Neg	Neg	41,190	Neg	Neg	ND	Neg	Neg	<40	Neg	Neg
2	4,072,969	NR	166	NR	ND	ND	124,125	Pos	Neg	<40	Neg	Neg	ND	ND	ND	340,330	Neg	Neg	<40	Neg	Neg	ND	ND	ND
3	368,533	NR	<40	R (new)	41	NR	446,782	Pos	Neg	<40	Neg	Neg	<40	Pos	Neg	6818	Pos	Neg	<40	Neg	Neg	<40	Neg	Pos
4^a^	322,952	NR	<40	NR	<40	NR	<40	Neg	Neg	<40	Neg	Neg	ND	Neg	Neg	<40	eg	Neg	720	Neg	Neg	<40	Neg	Neg
5	235,377	R (new)	<40	R (treated)	<40	NR	<40	Neg	Neg	756	Neg	Neg	<40	Neg	Neg	440	Neg	Neg	<40	Neg	Neg	<40	Neg	Neg
6	572,704	R (new)	64	R (treated)	ND	ND	78,008	Neg	Pos	ND	ND	ND	ND	ND	ND	<40	Neg	Neg	<40	ND	ND	ND	ND	ND
7	1650	R (new)	717	R (treated)	ND	ND	<40	Pos	Neg	<40	Neg	Neg	ND	ND	ND	862	Pos	Neg	<40	Neg	Neg	ND	ND	ND
8^a^	753,715	R (new)	433,030	R (treated)	202	NR	2977	Neg	Neg	ND	Neg	Pos	<40	Pos	Pos	4030	Neg	Neg	<40	Neg	Neg	2965	Neg	Neg
9	8841	NR	<40	NR	<40	NR	1659	Neg	Neg	<40	Neg	Neg	<40	Neg	Neg	<40	Neg	Neg	<40	Neg	Neg	205	Neg	Neg

Number 1 had V179D mutation at baseline which conferred low level resistance to efavirenz, nevirapine, rilpivirine and etravirine. Number 4 had G48S, I50R and I54R mutations at baseline which conferred potentially low level resistance to lopinavir/ritonavir, atazanvir/ritonavir and indinavir/ritonavir, intermediate resistance to nelfinavir and high level resistance to saquinavir/ritonavir. Number 8 had no mutation at baseline but at month 12 developed M184V, V106I, Y188I and P225HLP mutations. His regimen was changed to tenofovir, emtricitabine, darunavir/ritonavir at month 12. Grey cells represent detectable HIV RNA at month 12 or month 24 after antiretroviral therapy. BL, baseline; M, month; GC, gonorrhoea; CT, chlamydia; Ureth, urethral; R, reactive; NR, non‐reactive; ND, not done; Pos, positive; Neg, negative; STIs, sexually transmitted infections.

a These participants had resistant mutations found in their blood during the study period.

### 3.6 Risk behaviours and STIs at months 12 and 24 after ART

Of 117 participants at month 12 and 76 participants at month 24, 16.7% and 15.9% reported unprotected sex, and 20.9% and 17.2% had multiple sex partners respectively. Incidence rates were 13.4 per 100 person‐years for syphilis, 16.4 per 100 person‐years for gonorrhoea and 18.1 per 100 person‐years for chlamydia.

## 4 Discussion

We demonstrated a very high prevalence of STIs at baseline among Thai MSM who were newly diagnosed with HIV infection. More importantly, correlations between the presence of STIs and the detectability of HIV RNA in anogenital compartments were shown for the first time among Thai MSM prior to ART. The rates of STIs remained high at months 12 and 24 after ART but ART seemed to effectively suppress HIV RNA in all compartments. HIV‐positive Thai MSM in this study showed high acceptance of immediate ART at 93.0% and 95.6% at month 12 and 97.1% at month 24 achieved viral suppression of below 40 copies/mL after ART.

Syphilis prevalence of 16.1% among our Thai HIV‐positive MSM was much higher than the median prevalence among MSM in the South‐East Asian region of 1.2% (0.3% to 16.4%) 14 and among HIV‐positive MSM in Brazil (10.8%) 15, Switzerland (5.4%) 16 and the Netherlands (5.0%) 17. However, studies from Taiwan 18 showed an even higher prevalence of syphilis of 37.5% among HIV‐positive MSM. The rectum was the site most commonly infected with gonorrhoea and chlamydia in our cohort and other studies 15, 17. We found 23.1% prevalence of gonorrhoea which was higher than 1.5% to 5.2% reported from previous studies in HIV‐positive MSM 15, 16, 17, 19. Similarly, chlamydia prevalence of 32.8% in our cohort was also higher than 8.6% to 14.9% reported previously among HIV‐positive MSM 15, 16, 17, 19. Studies from Brazil and the US also found that MSM with HIV infection had significantly higher rates of STIs than HIV‐negative MSM 15, 20. Higher rates of STIs seen in our study than other series could be due to the lack of routine asymptomatic STIs screening and treatment among MSM in Thailand.

The correlation between STIs and anogenital HIV RNA, especially prior to ART and likely until HIV RNA is completely suppressed, points to the importance of integrating asymptomatic STIs screening into Treatment as Prevention programme for MSM. We found that HIV‐positive MSM who had any STIs were at a higher risk of having detectable seminal plasma HIV RNA before ART. This finding supports previous data from a case series in men which suggested that treatment of asymptomatic urethral STIs could reduce seminal plasma HIV RNA 10. In addition, detectable rectal HIV RNA among HIV‐positive MSM in our study was associated with having chlamydia at any sites, while its association with rectal gonorrhoea did not reach a significant level. Both chlamydia and gonorrhoea are known to induce anogenital inflammation and therefore can increase HIV RNA shedding in respective compartments. Our finding that chlamydia but not gonorrhoea was associated with detectable rectal HIV RNA is likely caused by a higher number of chlamydia than gonorrhoea among our participants which could provide a higher chance for any significant correlations to be revealed. One small study among US MSM reported that rectal HIV RNA was not correlated with rectal STIs but was correlated with plasma HIV RNA 11. High plasma HIV RNA was identified as a risk factor for detectable HIV RNA in seminal plasma, but not in rectal secretions, in our study. Interestingly, we found that HIV seroconverters tended to be more likely to have undetectable HIV RNA in anogenital compartments than those who were HIV positive at baseline. Low rates of STIs at seroconversions in our study might contribute to this finding.

HIV‐positive MSM continued to have a high burden of STIs after ART in our study. A recent systematic review identified that the prevalence of STIs among HIV‐positive individuals receiving ART was not different from untreated persons suggesting that STIs co‐infections could undermine efforts to use ART for prevention by increasing genital secretion infectiousness 9. A study among sexually active HIV‐positive MSM in the US also found that STIs and genital inflammation can partially override the suppressive effect of ART on seminal HIV shedding 21. We did not find anogenital STIs among participants who had detectable seminal or rectal HIV RNA after ART. The lack of association between the presence of STIs and anogenital HIV RNA detection after ART in our study, which differed from the US study, could potentially be explained by the inclusion into the US study of MSM within the first year of ART (20%), and therefore a higher proportion of MSM with detectable blood plasma HIV RNA (18%). This again pointed to the importance of asymptomatic STIs screening and treatment among HIV‐positive MSM prior to and during the first year of ART initiation. Our finding supports the confirmed efficacy of treatment as prevention seen among discordant gay couples with high STIs burden in the PARTNER and the Opposites Attract studies 22, 23. Data from a cohort of HIV‐positive MSM in London also showed that the presence of asymptomatic rectal chlamydia and gonorrhoea was not associated with increased rectal HIV RNA in those fully suppressed on ART 24. A recent systematic review and meta‐analysis of 4607 HIV RNA measurements from 2835 individuals also did not find a statistically significant effect of STIs on HIV RNA among individuals on ART 12. However, STIs transmission among virally suppressed MSM remains a concern which calls for action. The only case in our study who had detectable HIV RNA of >1500 copies/mL in blood at month 12 and in semen at month 24 was the case who developed resistance mutations over the first 12 months of ART. Support to ensure adherence to ART among HIV‐positive MSM who agree to start ART would not only benefit the suppression of HIV RNA in blood and anogenital compartments but also the prevention of drug‐resistant HIV transmission within the MSM community.

Our study is the largest cohort of HIV‐positive MSM in Asia where HIV RNA data could be determined in blood and anogenital compartments, both prior to and after ART. The first‐line ART regimen used in this study is the regimen currently recommended in the World Health Organization (WHO) Guidelines and therefore results have a high potential to be generalized for other countries. The detection of STIs in this study was through the use of NAAT in all participants, regardless of clinical symptoms or history, which should represent the true rate of STIs as most of these are asymptomatic 25. However, the use of NAAT for routine STIs screening will need to be balanced between the benefit and its high cost, and a study of cost effectiveness may be needed to guide the national policy. Some limitations identified in this study included the absence of HIV RNA data from HIV‐positive participants who did not accept immediate ART and did not have samples collected at 12 and 24 months after baseline. We found that 62% to 65% of MSM in our study had detectable anogenital HIV RNA at baseline which was in the range of 57% to 100% previously reported among men not on ART 26, 27, 28, 29. However, differences in anogenital sampling and HIV RNA measurement techniques which could affect the detection rate of HIV RNA in these compartments imply that direct comparisons of data between studies may not be simply made 27, 28. Our participants were not advised to abstain from sex prior to anogenital sampling and therefore we could not rule out the possibility of detecting HIV RNA in these compartments as a result of HIV RNA contamination from partners’ secretions through recent unprotected sex with an untreated HIV‐positive partner. Also, we did not take any history or perform laboratory testing to exclude herpes simplex virus infection or intra‐rectal recreational drug use which could cause ulcerative lesions/inflammation and potentially increase HIV shedding. However, none of the participants with detectable anogenital HIV RNA had symptoms/signs of ulcerations/inflammation recorded at that particular visit. Data in this study came from MSM who accessed services at highly experienced HIV testing and ART sites and might not represent MSM who attended services elsewhere in terms of HIV staging at diagnosis and ART adherence and associated HIV RNA suppression rate.

## 5 Conclusions

We confirmed a high prevalence of STIs before ART initiation and a high incidence of STIs after ART among HIV‐positive MSM. ART effectively suppressed HIV RNA in all compartments by 12 and 24 months. These findings support the importance of the scale‐up of treatment as prevention among MSM population globally. The correlation between STIs and anogenital HIV RNA, especially prior to ART and likely until complete HIV RNA suppression from ART is achieved, points to the importance of integrating asymptomatic STIs screening into Treatment as Prevention programme for MSM.

## Authors’ contributions

NP, CL and PPh designed the study. NP led the study and wrote the first draft of the report. SPa and TP performed laboratory testing. NP and DT designed the analysis. DT analysed the data. SN, JJ, SPe and TS coordinated the study and oversaw data management. NT, PC, PPa, ST, KPa, SS and KT implemented the study at their sites. KPu conducted study monitoring. All authors critically reviewed and approved the manuscript.

## Funding

National Research Council of Thailand, National Health Security Office program, Government Pharmaceutical Organization, Department of Disease Control, Ministry of Public Health, World Health Organization, Aids Fonds, and amfAR, The Foundation for AIDS Research.
